# Co-design of improved climbing bean production practices for smallholder farmers in the highlands of Uganda

**DOI:** 10.1016/j.agsy.2019.05.003

**Published:** 2019-10

**Authors:** E. Ronner, K. Descheemaeker, C. Almekinders, P. Ebanyat, K.E. Giller

**Affiliations:** a Plant Production Systems, Wageningen University, P.O. Box 430, 6700 AK, Wageningen, The Netherlands; b Knowledge, Technology and Innovation, Wageningen University, P.O. Box 8130, 6700 EW, Wageningen, The Netherlands; c International Institute of Tropical Agriculture, P.O. Box 7878, Kampala, Uganda

**Keywords:** *Phaseolus vulgaris*, Legumes, Participatory, Multi-criteria

## Abstract

We evaluated the usefulness of a co-design process to generate a relevant basket of options for climbing bean cultivation in the context of a large-scale project. The aim was to identify a range of options sufficiently diverse to be of interest for farmers of widely-different resource endowment. The co-design process consisted of three cycles of demonstration, evaluation and re-design in the eastern and southwestern highlands of Uganda in 2014–2015. Evaluations aimed to distinguish preferences of farmers between the two areas, and among farmers of different gender and socio-economic backgrounds. Farmers, researchers, extension officers and NGO staff re-designed treatments for demonstrations in the next season. Climbing bean yields and evaluation scores varied between seasons and sites. Evaluation scores were not always in line with yields, revealing that farmers used multiple evaluation criteria next to yield, such as marketability of varieties, availability of inputs and ease of staking methods. The co-design process enriched the basket of options, improved the relevance of options demonstrated and enhanced the understanding of preferences of a diversity of users. Developing options for resource-poor farmers was difficult, however, because they face multiple constraints. The basket of options developed in this study can be applied across the East-African highlands, with an ‘option-by-context’ matrix as a starting point for out-scaling. The study also showed, however, that consistent recommendations about the suitability of technologies for different types of farmers were hard to identify. This highlights the importance of a basket of options with flexible combinations of practices rather than developing narrowly specified technology packages for static farm types.

## Highlights

• Farmers, researchers and other stakeholders co-designed climbing bean options.
• Preference for options differed between farmers of different wealth and gender.
• Farmers' multiple evaluation criteria were incorporated in technology re-design.
• Finding suitable alternatives for resource-poor farmers remains challenging.
• Co-designed basket of options is applicable across the East African highlands.

## 1 Introduction

Agronomic research on experimental research stations has been successful in improving crop yields under favourable environments (Chambers and Ghildyal, 1985; Darnhofer et al., 2012). Yet the technologies developed often performed poorly on smallholder farmers' fields due to the heterogeneity of soil fertility and crop management (Zingore et al., 2007). Moreover, adoption was limited as the technologies developed were not suited to the needs, preferences and resource constraints of smallholder farmers (Collinson, 2001; Darnhofer et al., 2012; Vanlauwe et al., 2016). This led to the realisation that a better understanding of the context in which smallholder farming takes place was needed, and that farmers, the users of technologies, needed to be engaged in the technology development process (Chambers et al., 1989; Collinson, 2000; Nagy and Sanders, 1990). The 1980s witnessed the advent of farming systems research and participatory research. These approaches helped to improve researchers' understanding of farming systems, and to identify users' preferences, objectives and constraints for locally suitable technologies (Biggs, 1990; Chambers et al., 1989; Defoer, 2002).

The “transfer of technologies” paradigm evolved into an interactive and participative paradigm, whereby farmers are not simply considered receivers of technologies through researchers, but as partners in the design of new technologies (Almekinders and Hardon, 2006; Defoer, 2002; Klerkx et al., 2012). Moreover, farmers are increasingly recognized as designers of their own production systems (Prost et al., 2018; Salembier et al., 2018), whereby the role of agricultural researchers changes into supporting farmers' design and experimentation (Kraaijvanger et al., 2016; Le Gal et al., 2011; Prost et al., 2018). Adaptive and iterative approaches are advocated, which not only focus on the development of new technologies (Prost et al., 2018), but also include their implementation to better evaluate technology relevance and re-design when necessary (Martin et al., 2013; Meynard et al., 2012).

A conceptual framework that gives practical guidance to the (participatory) design of technologies is the Describe-Explain-Explore-Design (DEED) cycle (Descheemaeker et al., 2016; Giller et al., 2011). In the DEED cycle, a range of methods is applied to describe the current system, explain problems and opportunities for improvement, explore the implications and trade-offs of these opportunities and to design relevant options for new cropping or farming systems. Central to the DEED cycle is the co-learning between researchers, farmers and other stakeholders (Descheemaeker et al., 2016). Most studies applied the DEED cycle once (Franke et al., 2014; Rufino et al., 2011; Tittonell et al., 2009). Few studies have also considered the implementation of the designed options, with an iterative application of the cycle to allow farmers to test the options, provide feedback on them and to be engaged in the re-design of options to enhance their relevance. The studies that did (e.g. Dogliotti et al., 2014; Falconnier et al., 2017; Prost et al., 2018), all involved a relatively small number of farmers in a limited area.

Despite the increased recognition that including users' perspectives in the design process helps to develop locally relevant technologies, participatory approaches have therefore also been criticized for being time-consuming, site-specific and having limited potential for out-scaling (Conroy and Sutherland, 2004; Sumberg et al., 2003). This creates tension in an era where quick impact at scale is required by donors of agricultural research-for-development projects (De Roo et al., 2017; Glover et al., 2016). Approaches are therefore needed that take into account users' perspectives, while still producing results that can be used for out-scaling to larger groups of beneficiaries.

Out-scaling can be facilitated by the use of recommendation domains (Conroy and Sutherland, 2004; Descheemaeker et al., 2016); commonly based on agro-ecology, population density and market access (Farrow et al., 2016; Nelson and Coe, 2014; Wood et al., 1999). Socio-economic factors such as access to land, labour and capital also determine farmers' propensity to apply technologies (Doss, 2006; Feder and Umali, 1993; Sumberg, 2005), but are usually not considered in technology design and out-scaling (Collinson, 2001; Vandeplas et al., 2010).

In this study, we aimed to develop relevant technology options for a diversity of farmers through the application of an iterative co-design process. We applied this process in the context of a large-scale “research-in-development” project, focusing on the dissemination of grain legumes in 11 countries in sub-Saharan Africa. We zoomed in on one of these legumes in Uganda: climbing beans. Climbing beans offer potential for sustainable intensification of farming systems as, compared with the more widely grown bush bean varieties, climbing beans have a larger yield potential (up to 4 to 5 tons ha^−1^), biomass production and nitrogen-fixing capacity (Bliss, 1993; Ramaekers et al., 2013; Wortmann, 2001). Improved varieties of climbing bean were introduced in Rwanda in the 1980s (Sperling and Muyaneza, 1995) and spread to neighbouring countries including Uganda. In parts of southwestern Uganda climbing beans are now widely grown, but in other areas of the country they are still relatively new. Inclusion of climbing beans on the farm requires a change in cropping system (from maize + bush bean intercropping to sole cropping of climbing beans), and requires additional investments in staking material (Ronner et al., 2018b). Climbing beans can be seen as a ‘complex’ technology, consisting of multiple components/practices such as variety use, inputs, staking and other management practices (Ronner et al., 2018a). Given the required changes in cropping system and possible variation in the combination of practices, climbing beans make an interesting case for the application of a co-design process and the development of options for farmers with different opportunities and constraints.

The objectives of this study were to evaluate the usefulness of an iterative co-design process in generating a relevant basket of options for climbing bean cultivation for a diversity of farmers, and to develop an out-scaling tool for use of these options in the context of a large-scale project. We hypothesized that the co-design process would lead to options that could not have been developed by either farmers or researchers alone, and to relevant options for different types of farmers given their specific resource constraints. We first describe the co-design process, consisting of three cycles of demonstration, evaluation and re-design of practices. Next, we focus on options for different types of farmers. Finally, we present the basket of options and out-scaling tool that resulted from the co-design process.

## 2 Methodology

### 2.1 Study areas

The study was conducted in Kapchorwa District in eastern Uganda, located between 34.30° and 34.55° East and 1.18° and 1.50° North, and Kabale and Kanungu Districts in southwestern Uganda, located between 29.60° and 30.30° East and 0.35° and 1.50° South. The study sites are situated in the highland areas of Uganda, around 1800–1900 masl. We will refer to the study areas as the eastern and southwestern highlands. Annual rainfall in the eastern highlands averages 1600 mm and in the southwest 1100–1200 mm, falling in two rainy seasons: a long season from March to July (season A) and a shorter season from September to December (season B). Main crops in the eastern highlands are coffee, banana and maize (intercropped with bush bean), and in the southwestern highlands beans (climbing and bush beans), banana, maize, Irish and sweet potato. The eastern highlands have better access to markets, a larger population density, a shorter history of climbing bean cultivation and poorer access to staking materials than the southwestern highlands (see Ronner et al. (2018a) for more detail). Together, these differences represented the geographical context for the development of relevant options.

### 2.2 Co-design process and data collection

**Fig. 1:**
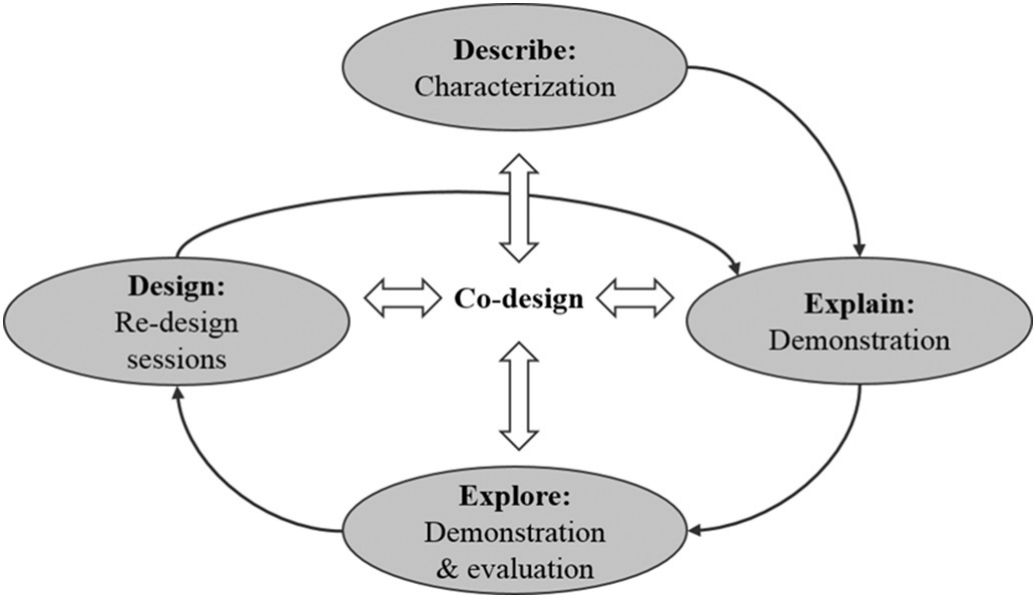
Iterative cycles of the co-design process, following the Describe-Explain-Explore-Design (DEED) cycle.

The co-design process consisted of three iterative cycles, following different phases of the DEED cycle (Fig. 1). A characterization of the two study areas was performed to Describe farming systems, climbing bean cultivation and socio-economic characteristics of households. The characterization helped to develop an initial set of practices for demonstrations to Explain current climbing bean cultivation and to Explore alternative practices. The Explore step also included farmers' evaluations of the practices. In the Design step we held sessions with a range of stakeholders to re-design practices for the next cycle.

#### 2.2.1 Describe: characterization

The study started in the eastern highlands during the first rainy season of 2014 (season 2014A) with a rapid characterization of the area through transect walks and interviews (*n* = 21) with key stakeholders including farmers, extension officers, representatives of farmers' organizations and NGO staff. The interviews aimed to establish the current role of climbing beans in the farming system, the extent to which farmers already cultivated them, and the most important production constraints. Farmers in two sub-counties of the eastern highlands (where farmers were least familiar with climbing beans) had already been introduced to on-farm try-outs of climbing beans with four treatments in 2013. Informal feedback on these try-outs was captured. The rapid characterization also included a participatory wealth ranking of households per sub-county, with people who were well-informed about the diversity of households in the community (e.g. teachers, community health workers, local government representatives, extension officers and farmers) (Bellon, 2001). Variables used to categorize households included farm size, number and type of livestock, type of housing, education (of children), type of employment and production orientation.

The rapid characterization was followed by a baseline survey (*n* = 75), which was also conducted in the southwestern highlands (*n* = 50), with questions related to household characteristics, agricultural production and legume cultivation and marketing (Stadler et al., 2016). Based on distinguishing criteria such as landholding, livestock ownership, type of housing, valuable assets, production orientation and most important sources of income – variables often used in other typology work in East-Africa as well – households were grouped manually into four farm types (see Ronner et al., 2018b, for a more detailed description). Four farmers from each type in both regions (32 in total) were selected for a detailed farm characterization, which contributed to the understanding of current cultivation of climbing beans and opportunities and constraints for different types of farmers (Marinus, 2015). In the southwest, transect walks and interviews (*n* = 12) were conducted after the baseline survey at the start of season 2014B. The rapid characterization and detailed follow-up were used as qualitative input to identify opportunities for the improvement of climbing bean production, taking into account region- and farm-type-specific challenges. The opportunities were translated, in consultation with key stakeholders, into a number of relevant treatments for different types of farmers for the demonstrations in the first co-design cycle. The detailed farm characterization also served to quantify the effects of different opportunities for the farm types in Ronner et al. (2018b).

#### 2.2.2 Explain & explore: demonstrations

Parallel to the characterization, we started the demonstrations for the first co-design cycle in the eastern highlands in season 2014A. Demonstrations in the southwestern highlands began in season 2014B. Demonstrations designed per region for season 2015A were repeated in season 2015B, to assess the performance of the same practices over two seasons. Demonstrations were held at parish level and were established on visible locations (road junctions, close to schools/churches), on homogeneous sites with not too steep slopes. The demonstrations were combined with so-called adaptation trials, in which larger numbers of farmers tried out practices on their own field, and also reflected on their performance (see Ronner et al., 2018a). The number of demonstrations held in the eastern highlands was 7 in 2014A, 8 in 2014B, and 7 in both 2015A&B; in the southwest 5 in 2014B, 15 in 2015A and 10 in 2015B. Each demonstration had a maximum of 12 treatments, and compared varying combinations of varieties, inputs (manure, mineral fertilizer) and staking methods. A summary of the treatments per season and region is found in the Supplementary material, Table S1. The initial design and the adjustment of the treatments after each co-design cycle is described as part of the results.

Demonstrations were planted on farmers' fields. Plot sizes in 2014A measured 10 × 10 m, but were decreased to 5 × 5 m from 2014B onwards as it was difficult to find large enough fields considering the small farm sizes in the densely populated highlands. Grain yields of the demonstrations were measured as unshelled, air-dry weights from sub-plots of 3 × 3 m in 2014A and whole plots from 2014B onwards. Only in 2014A a sub-sample of pods was shelled and oven-dried to establish dry grain weights. An average ratio of 0.7 between unshelled and shelled yields was found, which was applied to the rest of the data. Moisture content was highly variable, so all reported yields from the demonstrations represent air-dry, shelled grain yields.

#### 2.2.3 Explore: evaluations

Evaluations of the demonstrations were carried out in seasons 2014A, 2014B and 2015A. The evaluations served to identify which treatments farmers preferred, and to understand the reasons for preference. We aimed to distinguish preferences of farmers between the two geographical regions (eastern and southwestern highlands), and of farmers of different gender and socio-economic backgrounds. In 2014A, evaluations were carried out in four sub-counties in the eastern highlands, with six groups of farmers identified during the participatory wealth ranking: men and women from low (LRE), medium (MRE) and high (HRE) resource endowed households. For each group, five to eight people were identified for participation in the evaluations. The six groups evaluated the treatments separately, four times during the season (planting, staking, podding, harvest), by pairwise comparison and based on consensus. At each pairwise comparison, groups were also asked for their reasons for preference as an open question.

The evaluations in 2014A were logistically challenging and time-consuming. In search for a more easily applicable method for large-scale projects, demonstrations in season 2014B were evaluated only once during a field day (at podding of the beans). Evaluations were no longer done by the six groups, but by individual farmers visiting the field day. Scoring was used instead of pairwise comparison to facilitate statistical analysis. Farmers were also asked to fill in a reason for their score. To differentiate scores and reasons between different types of farmers, we also asked farmers to fill in gender, standard of living (better, same or worse as others in the village), and if they produced climbing beans mostly for home consumption or sale. Illiterate farmers were encouraged to ask help from staff or other farmers, but this method resulted in many missing or copied answers.

The evaluations in 2014B were easier, but information on farm types to disaggregate (reasons for) preference of treatments was less useful compared with 2014A. In 2015A we therefore evaluated the demonstrations with a random selection of farmers who participated in the adaptation trials, and from whom household characteristics were recorded (Ronner et al., 2018a). Farmers visited the demonstration trial during the season, but evaluated the treatments only once, after the season. Evaluation sessions were organized per sub-county. In each session, yields obtained in the demonstrations and estimates of input and labour costs calculated by researchers were presented. Farmers individually scored the performance of the treatments on a range of criteria (yield, variety traits, costs, labour, availability, etc.). These criteria were based on frequently mentioned reasons during the evaluations in previous seasons. To cross-check the relevance of these criteria and their relative importance, farmers were also asked to judge the criteria as “important”, “somewhat important” or “not important” (Bellon, 2001).

#### 2.2.4 Design: re-design sessions

At the end of seasons 2014A and 2014B, the evaluations formed the basis for re-design sessions per sub-county, in which farmers, researchers, extension officers and NGO staff re-designed treatments for demonstrations in 2014B and 2015A respectively. Participating farmers were selected by researchers and extension staff for their experience with climbing bean cultivation, innovativeness and involvement in the community. The re-design sessions were facilitated by researchers. At the end of season 2014A we used a goal-oriented approach with back-casting (De Graaf et al., 2009; Robinson et al., 2011), to explore opportunities to improve climbing bean yields for different types of farmers. Participants estimated current ‘best’ yields (in bags per acre) achievable by HRE, MRE and LRE households as a starting point. We then explored opportunities to improve these yields through back-casting: to identify which steps (practices) are needed to reach the best yields for the different farm types. Researchers translated the practices suggested during the re-design sessions into treatments for the demonstrations, and ensured that treatments were sufficiently replicated across sub-counties.

The re-design sessions in 2014B were informed by the results of the detailed characterization research performed in the season before, by the analysis of the yields of the different treatments and by farmers' evaluations of treatments during the field day. Hence, more information on opportunities and constraints for climbing bean cultivation was available than after the first season. The session was therefore narrowed down to discuss the performance of the different treatments, the field day evaluations and the reported challenges, followed by a direct focus on the development and improvement of practices for different types of farmers for season 2015A. No re-design session was held for season 2015B, as the aim was to assess performance of the same practices over two seasons.

### 2.3 Data analysis

Statistical analyses were performed in RStudio Version 1.0.143 (R Core Team, 2017). Differences in yield between the treatments in the demonstrations were analysed per season and region with a linear mixed model with treatment (variety, input, staking method) as fixed and farm as random factor. In season 2015B, two plot yields of >6000 kg ha^−1^ were considered unrealistically large and were removed.

Evaluation methods, and therefore data analysis, differed between seasons. In 2014A, a matrix with each pairwise comparison of treatments per group and growth stage was constructed. Per comparison, a score of 1 was assigned to the treatment that was preferred, and a score of −1 to the treatment that was not preferred. When the group could not reach consensus (1% of comparisons), a 0 score was given. The average of these scores over all groups and growth stages resulted in an overall ranking of treatments. Differences in preference for treatments between gender and wealth groups were assessed through an analysis of deviance from the overall ranking (Coe, 2002). For this, each pairwise comparison received a binary value: 1 if the preferred treatment in the comparison complied with the overall ranking of treatments, and 0 if the preference did not comply. Differences in compliance with the overall ranking were assessed with a binomial generalized linear mixed model, with gender and wealth as fixed and group ID as random factor. Reasons for preference of treatments, asked as open question per group, were categorized. Per treatment, the number of times a certain category was mentioned was counted and divided over the total number of reasons given for that treatment.

In 2014B, original individual evaluation scores between 1 (very good) and 5 (very poor) were converted to a score between 1 and −1. Differences in scores between treatments and regions were assessed with a linear model. Because of an interaction between treatment and region influencing the scores, differences in scores between groups of different gender, standard of living and production orientation were analysed with linear models per region. Reasons for preference of treatments were considered unreliable because of the many missing and copied answers and were not analysed.

In 2015A, farmers' scoring of the performance of the treatments on each criteria was combined with the perceived importance of this criterion to obtain an ‘attainment index’ for each treatment, ranging from 1 to −1 and indicating how well a treatment met all the criteria valued by farmers (Bellon, 2001). A detailed description of the development of this attainment index is given in the Supplementary material, S2. Differences in scores between regions, treatments and groups with different household characteristics were analysed with a linear mixed model, with group ID as random factor. Household characteristics included were: gender (0 = female, 1 = male), education of the farmer (0 = none or primary, 1 = secondary or higher), age of the household head, farm size (ha), number of livestock, months of food security (0 ≤ 10 months, 1 = 10–12 months), production orientation (0 = all or most farm produce consumed, 1 = half or most farm produce sold), off-farm income (0 = all income from farming, 1 = some to most income from off-farm activities), frequency of hiring labour (0 = never or sometimes, 1 = regularly or permanently) and income from salary, pension or remittances (0 = no, 1 = yes). For this analysis, an outlier in farm size of >20 ha in the southwestern highlands was removed. Differences in importance of criteria (1 = very; 0 = somewhat; −1 = not important) were analysed with ordinal logistic regression. A cumulative link model (clm) in the package *Ordinal* was fitted with the default ‘logit’ link function.

## 3 Results

### 3.1 Characterization of the study areas and design of first options

At the start of the study, about 10% of farmers in the eastern highlands cultivated climbing beans, in the southwestern highlands about 50%. From the farmers who grew climbing beans, 75% intercropped climbing bean with banana and/or coffee in the eastern highlands. In the southwestern highlands sole cropping was more popular. In both regions, the use of inputs in climbing beans was low: in the eastern highlands 25% (2 out of 8 farmers) grew climbing beans with DAP fertilizer and none of them used organic fertilizer. In the southwestern highlands none of the farmers cultivating climbing bean used mineral fertilizer, but 34% used organic fertilizer. Women or both men and women managed and took decisions about sale of climbing beans, with men playing a slightly larger role in the southwestern than eastern highlands. In both regions, women had a relatively larger role in the crop management of the beans, and men in decisions about sale. Perceived constraints for climbing bean cultivation were staking and the additional labour demand in the east, and rats, birds and poor soil fertility in the southwest (Marinus, 2015).

Demonstrations started in the eastern highlands only, in season 2014A. The staking challenge in the east was taken as the basis for the design of the first demonstrations in this area. An improved climbing bean variety (NABE 26C) was planted with manure and TSP fertilizer and different staking methods: 1) the commonly used single wooden stakes; 2) a low-cost alternative in the form of ropes of banana fibre and sisal strings tied to a wooden frame and 3) tripods (three wooden stakes tied together). The latter were expected to enhance yields, as they would prevent the stakes from falling over under the weight of the beans. Although tripods increase labour at staking, they could reduce labour need during the season. Next to staking methods, the demonstrations also compared the improved variety with a local variety with and without manure and TSP.

### 3.2 Iterative co-design cycles

#### 3.2.1 First cycle (season 2014A-2014B)

##### 3.2.1.1 Demonstration and evaluation

**Fig. 2:**
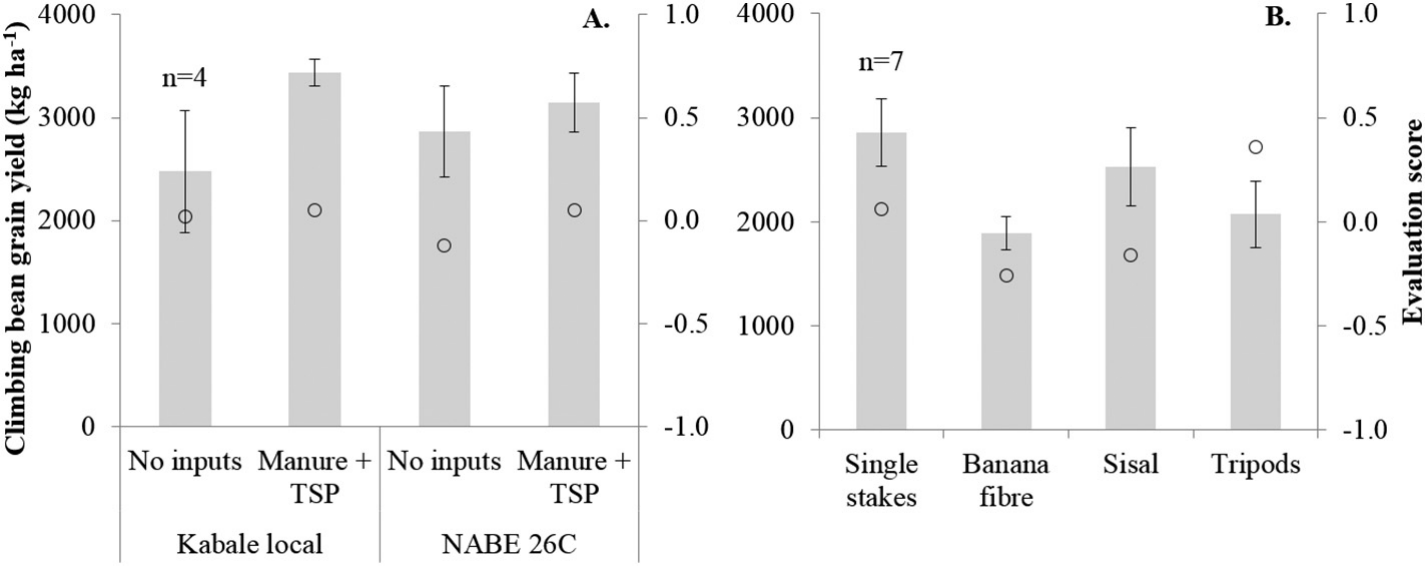
A&B: Climbing bean grain yields (bars, primary y-axis) and evaluation scores (circles, secondary y-axis) for varieties and inputs (A) and staking methods (with variety NABE 26C with manure + TSP) (B) in demonstrations in the eastern highlands of Uganda, season 2014A. Error bars represent standard error of the mean, n = number of demonstrations per site.

Grain yields in the demonstrations did not differ between varieties, but the application of manure + TSP significantly increased yield compared with the treatment without inputs (Fig. 2A). There was no interaction between varieties and inputs. Farmers preferred the treatments with inputs over the treatment without inputs, and this preference was more pronounced for variety NABE 26C, although the difference in yield was smaller than for variety Kabale local. In the demonstration of staking methods, yields of banana fibre were significantly smaller than for single stakes (Fig. 2B). Partly, this could be related to stake length: banana fibre and sisal strings were shorter (137 cm) than single stakes and tripods (215 cm). Tripods did not yield better than single stakes, but farmers ranked tripods first.

##### 3.2.1.2 Re-design

In the re-design sessions (*n* = 4), stakeholders considered single stakes, sisal strings and tripods all as appropriate staking methods for good yields. Banana fibre was thought to break easily, although it was considered an option for poorer farmers. Banana fibre could be made stronger by braiding, but this was perceived to be laborious. Stakeholders would recommend TSP or DAP fertilizer (cheaper and easier to access than TSP at the time of study) to HRE farmers who could afford it to obtain the ‘best’ yields identified; manure was suggested to achieve the target yields for medium and poorer farmers. Other than that, it proved difficult to develop suitable or innovative options for poorer farmers. Rather, stakeholders would advise poorer farmers to do everything the same as farmers with the largest yields, but on a small piece of land to reduce costs.

The evaluations and re-design sessions determined the design of the demonstrations in season 2014B (Table 1). An important change was the wooden frame for the banana fibre ropes and sisal strings. Farmers found the poles and rafters needed for this frame too expensive. Based on a suggestion from a farmer participating in re-design sessions (who had tested this option himself), the number of poles and rafters was reduced by half, and two rows of strings instead of one were hung down from one rafter. Researchers related the poorer yields of this staking method to the height of the frame, and ensured that the frame was taller than in the first season. Stakeholders also jointly decided to change the number of seeds per hole in the demonstrations from one to two. Farmers preferred to plant three to four (or more) seeds per hole because of experiences with poor germination on poor soils. Researchers suggested that seed that did not germinate would have to be replanted (‘gap filling’), but farmers found this too laborious. Farmers agreed, however, that planting many seeds per hole resulted in a loss of seed and overloading of stakes. Two seeds per hole was considered a good compromise between reducing the risk of poor germination and avoiding additional labour for gap filling.

**Table 1:** Actions, reasons and information sources for re-design of the demonstrations in seasons 2014B and 2015A in the eastern and southwestern highlands of Uganda. Region-specific actions are specified with EH (eastern highlands) or SWH (southwestern highlands).

Re-design action	Reason	Source
*Demonstrations 2014B*		
Banana fibre ropes no longer demonstrated, only sisal strings	Poor results of banana fibre. Farmers could adapt the method to banana fibre	All stake-holders
Wooden frame for banana fibre ropes and sisal strings adjusted	Cost reduction	Farmer
Variety NABE 26C replaced by NABE 12C	Available in larger quantities	Researchers
Variety Kabale local and NABE 12C both demonstrated	Preference for varieties differed between groups	All stake-holders
Variety Kabale local no longer demonstrated on strings	Variety was considered too heavy and leafy for strings. Tripods were considered particularly suitable for this variety	All stake-holders
Number of seeds per hole increased from one to two	Compromise between farmers' practice of large number of seeds per hole (reducing risk of poor germination) and researchers' practice of one seed per hole and ‘gap filling’ of seed that did not germinate	All stake-holders

*Demonstrations 2015A*		
Comparison of TSP with DAP and DAP+NPK (EH)	Comparison of new fertilizer TSP with commonly used DAP and DAP + NPK	Farmers
Strings still demonstrated despite small evaluation score (EH)	Frame and strings considered expensive, but still wanted to evaluate performance	All stake-holders
Tripods no longer demonstrated (SWH)	Beans did not receive enough sunlight and aeration, affected by blight	Farmers
Comparison of row planting and broadcasting (SWH)	Row planting was expected to reduce damage of rats	NGO staff, extension
Removing growing tip of beans at 1.80 m (SWH)	Avoid shade, enhance podding	Farmers
Comparison of local variety with (multiple) improved varieties (both regions)	Farmers preferred improved varieties for seed size, taste and maturity time but wanted comparison with local varieties	All stake-holders

Demonstrations in the southwestern highlands, starting in 2014B, had the same design as the re-designed trials in the east to identify regional differences and preferences. The issue of poor soil fertility, identified as one of the main production constraints during the characterization in the southwest, was addressed through the different fertilizer treatments (Supplementary material, Table S1). In one district in the southwest, papyrus strings were included as treatment as alternative for sisal or banana fibre, as papyrus was widely available in this district.

#### 3.2.2 Second cycle (season 2014B – 2015A)

##### 3.2.2.1 Demonstration and evaluation

**Fig. 3:**
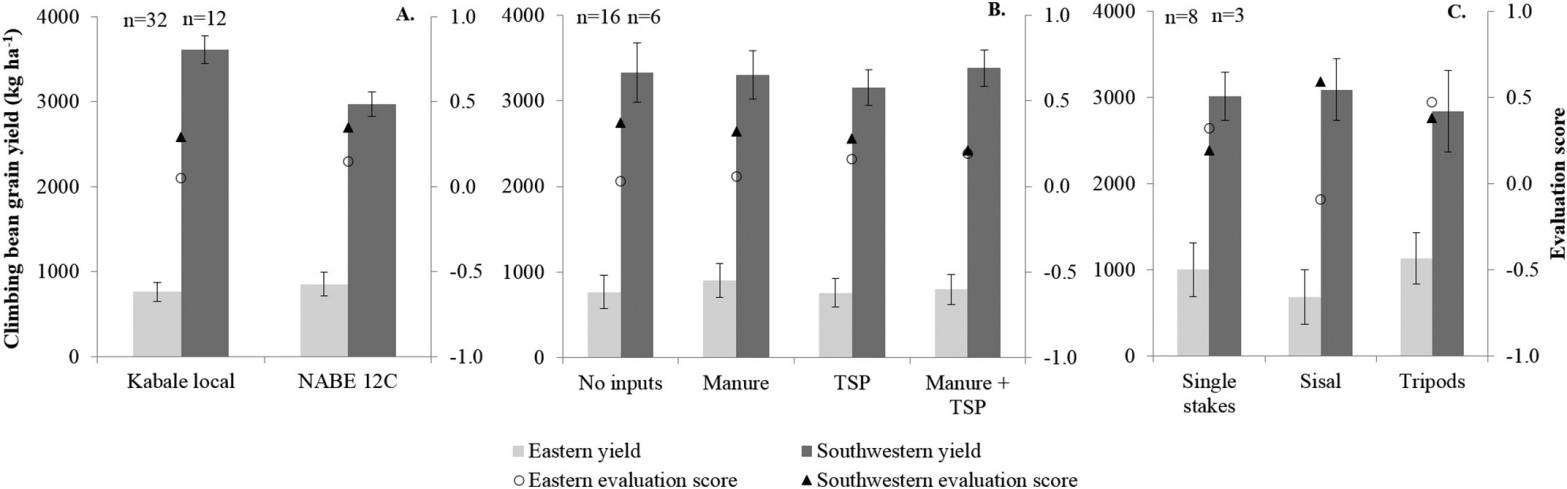
A, B & C: Climbing bean grain yields (primary y-axis) and evaluation scores (secondary y-axis) for varieties (average of all input treatments) (A), inputs (average of all varieties) (B) and staking methods (variety NABE 12C with manure + TSP) (C) in demonstrations in the eastern and southwestern highlands of Uganda, season 2014B. Error bars represent standard error of the mean, n = number of demonstrations per site.

Yields of the demonstrations in the southwestern highlands were much larger than in the eastern highlands (Fig. 3). For varieties, yields of Kabale local were significantly larger than for NABE 12C in the southwest (Fig. 3A). In both regions, however, farmers evaluated NABE 12C significantly better. The use of inputs did not affect yields (Fig. 3B). This may explain why farmers in the southwest gave the largest score to the treatment without inputs (although in the east, this treatment received the smallest score). Differences in yield between the staking methods were not significant, but farmers in both regions gave the largest score to the treatment with the largest yield: in the east to tripods, in the southwest to sisal strings (Fig. 3C).

##### 3.2.2.2 Re-design

The re-design sessions at the end of season 2014B in the eastern (*n* = 3) and southwestern highlands (*n* = 5) revealed differences between the two regions. In the east, the use of mineral fertilizer was much more common than in the southwest. Stakeholders in the east therefore had questions about the difference between the use of TSP and DAP fertilizer, and also mentioned that if they applied fertilizer to climbing bean themselves, they often applied DAP + NPK together. They wished to compare TSP, DAP and DAP + NPK. In the southwest, mineral fertilizer was rarely used and only applied to cash crops like Irish potato. Researchers suggested that with a rotation of Irish potatoes followed by climbing bean, the beans could benefit from the fertilizer applied to the potatoes and use the residual phosphorus. Stakeholders fed back that climbing beans grown after Irish potato do not do well (which could point at a problem of nematodes), so they commonly only grow climbing bean in rotation with sorghum or maize. The option of including the rotation with Irish potato over two seasons in the demonstrations was therefore not implemented.

Mineral fertilizer was still considered worth demonstrating as farmers would prefer this, when available, over scarce and bulky manure. Other particular issues highlighted in the southwest were problems with rats and birds. NGO and extension staff recognized this as a widespread problem and shared good practices such as clearing buffer zones around the field and planting in rows instead of broadcasting – the open spaces would scare the rats. To reduce bird damage, farmers were advised to plant all at the same time. Farmers in the southwest also mentioned that they were advised earlier on to cut the growing tip of the beans once they exceeded 1.80 m, which would avoid shade from the canopy and enhance podding. A comparison with non-cut growth was suggested.

In both areas, farmers were particularly interested in the comparison of varieties. They mentioned that advantages of the demonstrated varieties over their local varieties were for instance seed size, taste and maturity time. It was suggested to include local varieties in the demonstration, to compare their performance with the improved varieties. Variety NABE 26, which was included in the demonstrations in season 2014A, turned out to be affected by bean anthracnose. Researchers wanted to verify this diagnosis and included this variety as well. The re-design sessions therefore led to a number of joint ‘research questions’, which were tried to be answered through the comparison of treatments in the demonstrations (Table 1). Parallel to the demonstrations, researchers also initiated agronomic trials on the interactions between variety, input (manure + TSP) and stake length, and the contribution of nutrients other than P, considering the limited responses to manure and TSP fertilizer (data not presented). These trials served to inform the dissemination campaign in subsequent seasons.

#### 3.2.3 Third cycle (2015A and 2015B)

##### 3.2.3.1 Demonstration and evaluation

**Fig. 4:**
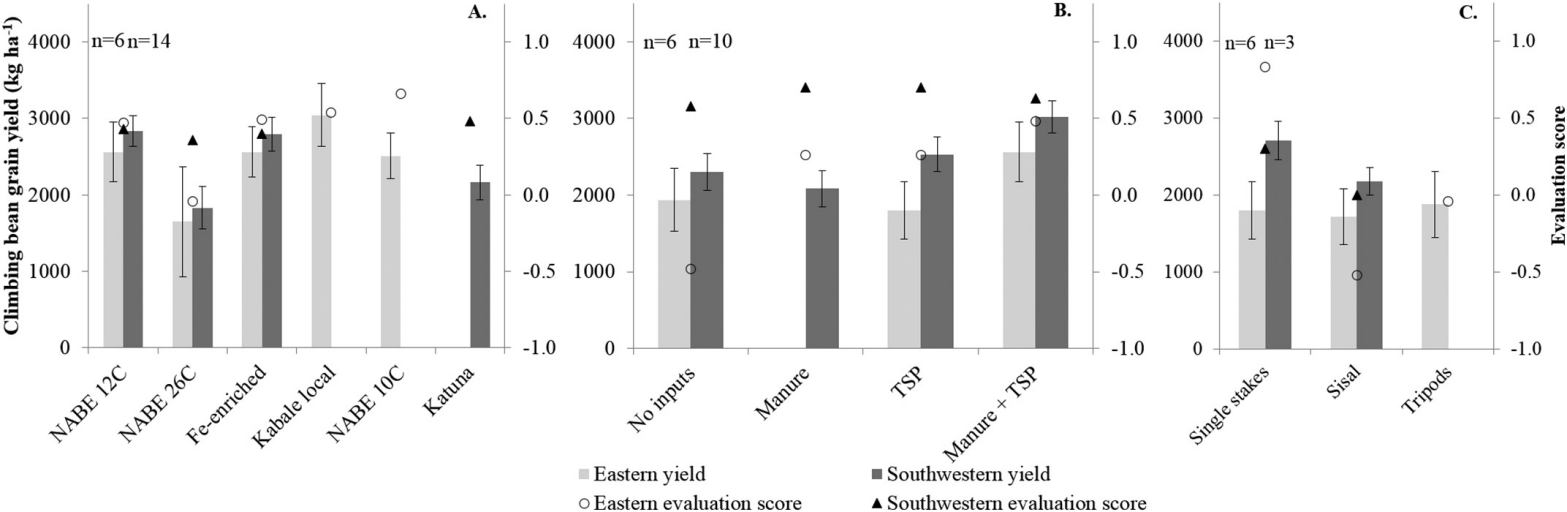
A, B & C: Climbing bean grain yields (primary y-axis) and evaluation scores (secondary y-axis) for varieties (with manure + TSP) (A), inputs (variety NABE 12C) (B) and staking methods (variety NABE 12C with TSP) (C) in demonstrations in the eastern and southwestern highlands of Uganda, season 2015A. Error bars represent standard error of the mean, n = number of demonstrations per site.

Yields in the southwestern highlands were again larger than in the eastern highlands in 2015A, but differences were smaller than in 2014B (Fig. 4). Varieties NABE 12C and Fe-enriched had larger yields than NABE 26C (Fig. 4A), but the difference was only significant in the southwest. Evaluation scores for NABE 26C were also significantly smaller than for the other varieties. Farmers in the eastern highlands gave the largest score to their ‘local’ variety NABE 10C. The use of inputs had a significant effect on yield only in the southwest: manure + TSP had a larger yield than the treatment without inputs (Fig. 4B). Conversely, evaluation scores for inputs did not differ in the southwest, but in the east manure + TSP received a significantly larger score than the treatment without inputs. The differences in yield between the staking methods were not significant. Farmers in both areas gave the largest score to single stakes (Fig. 4C). The comparison between TSP, DAP and DAP+NPK in the eastern highlands did not result in differences in yield (data not presented). In the southwest, row planting resulted in significantly larger yields than broadcasting; removing the growing tip of the climbing beans significantly reduced yields by >1 t ha^−1^.

The treatments in the demonstrations roughly stayed the same in season 2015B. Only variety NABE 26C, confirmed to be heavily affected by bean anthracnose in preceding seasons, was left out of the demonstrations. Some trends in yields in 2015B differed from 2015A: in the east, yields were significantly larger for variety NABE 12C than NABE 10C and for TSP and manure + TSP than for the treatment without inputs. In the southwest, differences between inputs were not significant. Broadcasting and removal of the growing tip of the beans had larger yields than row planting and unlimited growth, but differences were not significant.

##### 3.2.3.2 Re-design

The co-design process ended after season 2015B so no re-design session was held. After multiple seasons of co-design and farmers' testing of climbing beans in adaptation trials we still found, however, that 50–75% of the farmers in the eastern and some districts of the southwestern highlands continued intercropping of climbing beans with banana and/or coffee. The prevalence of intercropping was recognized from the start, but was difficult to include as a set of treatments due to huge variability in existing banana/coffee fields in terms of spacing and age of the plants. Considering the co-design goal to develop locally relevant options, however, we could not ignore the importance of this practice. Little is known about the effects and best management practices for climbing beans in intercropping, which also makes the topic worthy of investigation. We therefore set up a trial in seasons 2016A and 2016B to assess the effects of banana leaf pruning on light availability and climbing bean yields in intercropping, building on earlier work of Ntamwira et al. (2013). A local and an improved climbing bean variety were exposed to pruning of banana up to eight leaves. We expected enhanced light availability, resulting in larger climbing bean yields under pruning, and also expected differences between the two varieties as they may differ in their tolerance to shading. Pruning of banana significantly enhanced the fraction of PAR transmitted through the banana canopy, but did not show a significant effect on climbing bean yields. There was no difference in yield between the two varieties (for more details see Ronner et al., 2019).

### 3.3 Options for different types of farmers?

#### 3.3.1 Preference of treatments

Evaluation scores were disaggregated by gender and wealth groups, to identify relevant options for different types of farmers (data not presented). Varieties were generally evaluated similarly. Only in season 2014B, women farmers in the eastern highlands gave variety Kabale local a significantly larger score than men. They specifically liked the taste and indicated to use the leaves as vegetable. Inputs were generally valued by wealthier farmers: manure + TSP received significantly larger scores from HRE farmers 2014A, from farmers producing climbing beans mostly for sale in 2014B and from farmers with relatively large farms in 2015A. The treatment without inputs received significantly larger scores from LRE farmers in 2014A and from farmers producing climbing beans mostly for home consumption in 2014B. Although strings were introduced as low-cost alternative for poorer farmers, they received significantly larger evaluation scores by farmers mostly producing for sale in the east in 2014B, and by farmers with larger farms and hiring labour more often in the southwest in 2015A. However, farmers producing mostly for sale in the southwest preferred single stakes in 2015A.

#### 3.3.2 Reasons for preference

**Fig. 5:**
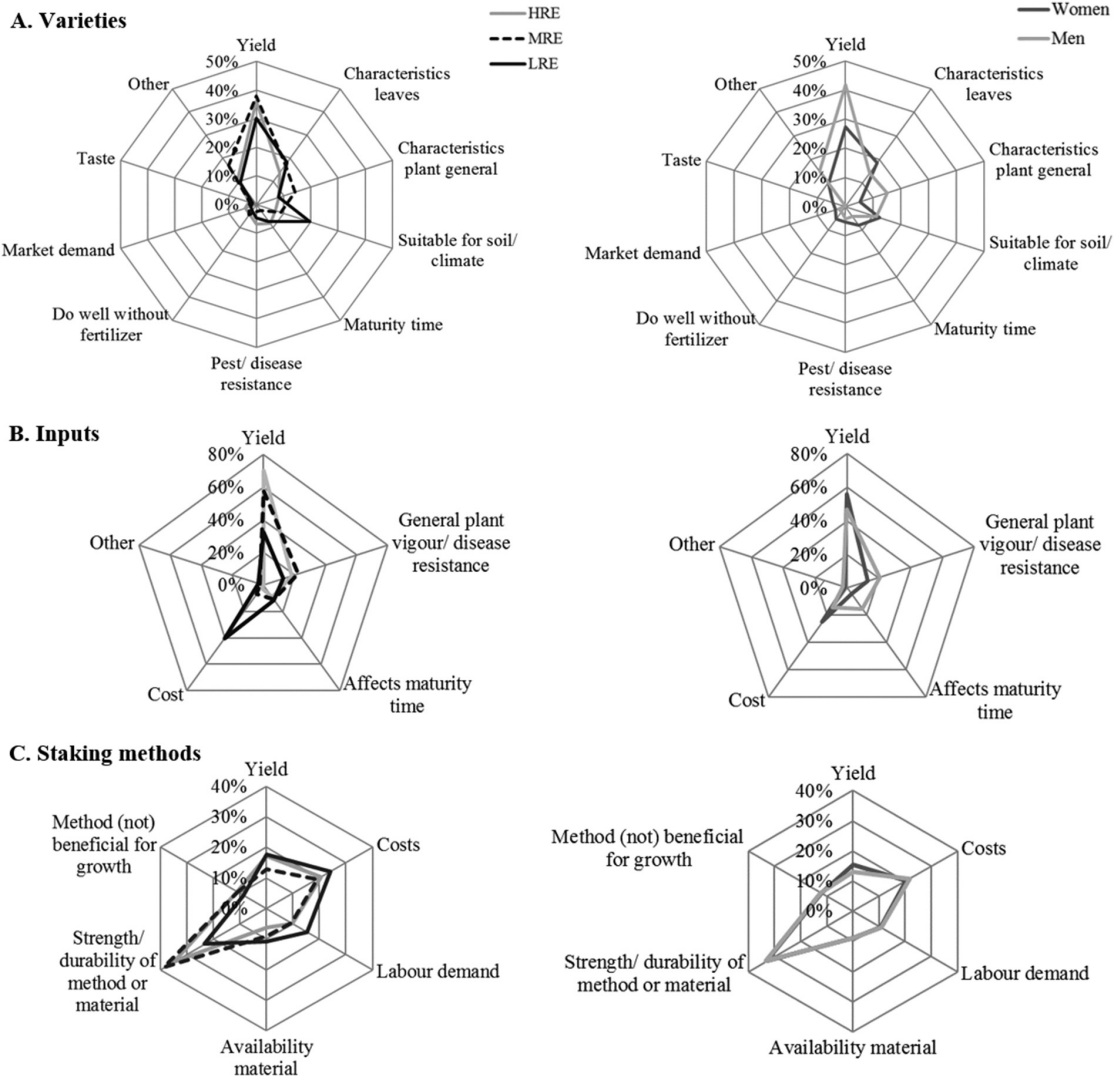
A, B & C: Reasons for preference of varieties (A), inputs (B) and staking methods (C) mentioned by farmers of low (LRE), medium (MRE) or high (HRE) resource endowment (left side) and men and women (right side) in pairwise comparison of treatments in the eastern highlands of Uganda, 2014A.

Reasons for preference may also differ between wealth and gender groups, which could explain their choices for a certain treatment. In 2014A, yield was the most frequently cited reason for preference of varieties (Fig. 5A). Other reasons were characteristics of the leaves (e.g. number, size, shape) and the plant in general (strong, healthy, tall). Reasons for preference hardly differed between groups; only yield seemed to be relatively more important to men than women. For inputs, yield was the most important reason for preference for HRE farmers whereas LRE farmers mentioned costs more frequently (Fig. 5B). Men and women largely considered the same reasons. For staking methods, all groups of farmers mentioned strength of the method (tripods) or durability of the material (sisal strings vs banana fibre) more often than yield (Fig. 5C). LRE farmers also mentioned costs more frequently than yield, and labour demand of the staking methods more frequently than MRE and HRE farmers. Men and women gave similar reasons for preference of staking methods.

In 2015A, farmers again rated yield as important criterion for evaluation, although for varieties, inputs and staking methods other criteria received larger scores such as marketability, disease resistance, availability of inputs, ease of the staking method and re-usability of staking material (Supplementary material, Table S3). The importance of criteria differed between regions: farmers in the eastern highlands found labour, the yield without fertilizer and the availability of inputs significantly more important; farmers in the southwest costs and marketability. Gender had no effect on the rating of importance. Relationships with household characteristics (age, education, farm size, livestock number, months food secure, production for home consumption or sale, income from off-farm activities, frequency of hiring labour) were variable. Farmers producing mostly for home consumption found costs and the benefit/cost ratio more important than farmers producing mostly for sale. Other relationships seemed contradictory (farmers with off-farm income minded less about costs, but found the yield without fertilizer more important; farmers with an income from salary, pension or remittances in the east found the yield of varieties without fertilizer less important, but their counterparts in the southwest attached less value to the yield with fertilizer); counterintuitive (farmers with smaller farms found the yield with fertilizer more important; farmers producing mostly for sale found the maturity time of varieties more important); or could not be explained well (farmers from households with older household heads found the yield with fertilizer and the strength of staking material less important; farmers with larger farms found disease resistance more important). Differences in reasons for preference therefore remained largely unexplained based on individual household characteristics in 2015A.

#### 3.3.3 Preference for co-designed versus researcher-designed options

Varieties NABE 10C and Katuna were introduced in the demonstrations of season 2015A at farmers' requests. These varieties received the largest evaluation scores in season 2015A, whereas yields of these varieties were not larger (in the southwest even smaller) than the newly introduced varieties (Fig. 4). Again, this demonstrates the importance of criteria other than yield. For instance, farmers gave variety NABE 10C the largest score for grain colour, maturity time and suitability for the climate, whereas NABE 12C was particularly valued for yield (with fertilizer) and grain size, but scored negatively for disease resistance. The same holds for variety Katuna in the southwest: the scores for insect tolerance and disease resistance were better for Katuna than NABE 12C, even though they had the same score for yield.

For inputs, the co-design process resulted in the inclusion of DAP and DAP+NPK in the demonstrations in the eastern highlands in season 2015A, next to TSP as the researcher best-bet option. Farmers gave the largest evaluation score to the treatment with DAP. In contrast, DAP+NPK received a negative score; only slightly better than the control. Both DAP and DAP+NPK got much better scores for the availability of inputs than TSP and manure. Currently, these inputs are also cheaper than TSP in the area. Farmers in the southwest gave manure or TSP alone larger scores than manure + TSP.

Among the staking methods, strings were included as low-cost alternative for poorer farmers. Strings consistently received the lowest scores, however, except in the southwest in 2014B. In both 2014A and 2015A, the availability of the material and the additional labour demand were seen as negative aspects of strings versus single stakes. In 2015A, the costs, ease of the method and re-usability of the material all received a negative score for strings. Single stakes, the researcher best-bet, received the largest score for yield and ease of the method. Tripods were particularly valued for the strength of the method, but received a smaller score than single stakes for all other criteria.

### 3.4 Basket of options and out-scaling tool

**Fig. 6:**
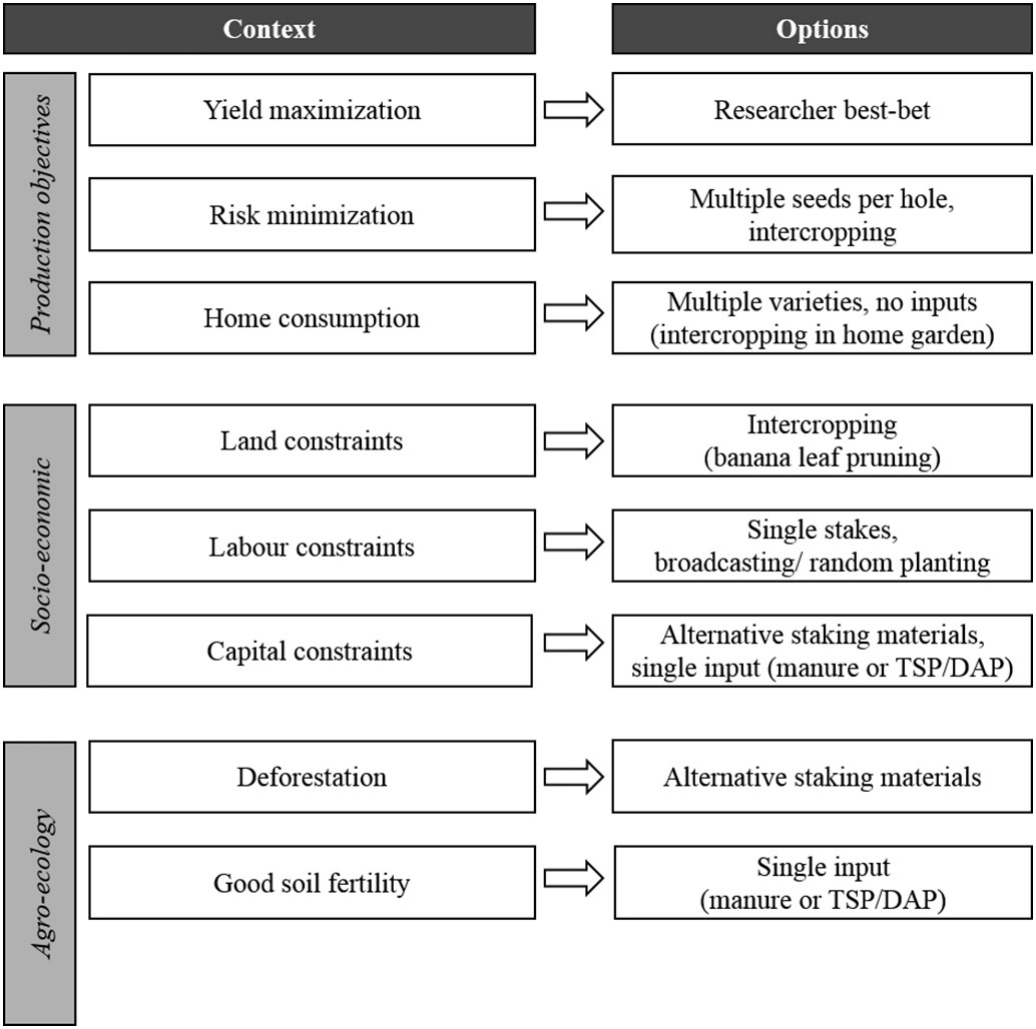
‘Option-by-context’ matrix as out-scaling tool for climbing bean cultivation, showing how researcher best-bet and additional options fit into certain contexts.

Through the iterative co-design process, the initial researcher designed practices were modified and new practices were added. This process led to the development of a basket of options (Table 2). Every season, options from this basket were added, refined or discarded. Such a basket of options could be used by other projects or development agents aiming to expand climbing bean cultivation to new areas. The identification of reasons for preference for certain options provided insight in the context in which farmers make choices. Certain options were preferred because of farmers' production objectives, their production constraints, or in the context of a certain agro-ecological environment or farming system. Hence, an ‘option-by-context’ matrix could serve as a guide for out-scaling and extension (Fig. 6). With a new project or expansion to new areas, a rapid characterization of the distinguishing context-factors is a first step. Options suitable for the context at hand can then be selected from the matrix. Such a matrix should not be seen as prescriptive, but provides guidance in the alternatives available to different types of farmers. Farmers' feedback on the experimentation with the options can also be used to refine the matrix.

**Table 2:** Researcher best-bet practices; additional options tested during the co-design process and reasons for preference (other than yield) of the additional options in the eastern and southwestern highlands of Uganda, seasons 2014A-2015B. Researcher and additional options resulting from the co-design process together form a basket of options for climbing bean cultivation.

	Researcher best-bet	Additional options	Reasons for preference
Varieties	Improved variety	Multiple varieties	Multiple variety traits
Inputs	Manure + TSP	No inputs	Costs
		Manure or TSP only	Availability, costs
		DAP	Availability, costs
Staking	Single stakes	Strings	Availability, costs
		Tripods	Strength, labour
	Wooden stakes	Banana fibre	Availability, costs
		Papyrus	Availability, costs
		Maize stalks	Availability, costs
		Sisal	Strength, re-usability, costs
		Nylon	Strength, re-usability, costs
	Stakes >1.75 m	Shorter stakes	Availability, control bird damage
Other practices	Sole cropping	Intercropping	Land scarcity, risk reduction
	Row planting	Broadcasting/ random planting	Labour
	One seed per hole	Two or more seeds per hole	Risk reduction, labour

## 4 Discussion

### 4.1 Lessons learned from developing and applying a co-design process

During the Description phase of the co-design process and the design of a first set of options to be included in the demonstrations, farmers were involved in a consultative way (Barreteau et al., 2010; Martin et al., 2013). This resulted in the demonstration of, among other practices, a low-cost staking method. After farmers evaluated the relevance of this method, it turned out that the initial design goal was not reached: farmers still considered the low-cost method too expensive. The subsequent collaboration with farmers resulted in the re-design of the method. The collaboration between farmers and researchers therefore helped to revise practices to better meet their goals. Sometimes the design goals themselves had to be redefined based on farmers' evaluation criteria: e.g. from “better yield” to “good marketability” for varieties, or from “low costs” to “easy method” for staking. These examples show the necessity of approaching design as an adaptive, iterative process of more than one cycle, and illustrate the inadequacy of the linear research-design-diffusion model (Meynard et al., 2012; Prost et al., 2018; Röling, 2009).

The adaptive approach also presented a trade-off: the re-design of the demonstrations each season allowed taking into account practices that farmers valued, but at the same time this meant that practices could not always be compared over multiple seasons or in both study areas. Scientific evidence supporting the decision for continuing or discarding a certain practice was therefore not always established. Salembier et al. (2018) recognize this dilemma in co-design processes and distinguish between “scientific knowledge production” and the “use of knowledge in a design process”, which both follow different rules. Knowledge generated during the co-design process often served to broaden explorations, and to stimulate the design by other stakeholders than researchers (cf. Bos et al., 2009; Salembier et al., 2018). Furthermore, even those treatments that could be compared over multiple seasons and sites showed a large variation in yield (in line with earlier findings on farmers' fields of Franke et al., 2016; Kraaijvanger et al., 2016; Ronner et al., 2018a). After four seasons, we only identified a significant effect on yield of manure + TSP compared with the treatment without inputs. This made it hard to draw firm conclusions and recommendations, even when scientific knowledge was produced.

The approaches used in the co-design process evolved from season to season. For instance, the different evaluation methods used were the result of a trade-off between the degree of detail and the ease of the method. The scoring method used in season 2015A was considered most useful as it allowed statistical analyses. However, with this method farmers judged most criteria as (very) important (Supplementary material, Table S3), which limited a distinction between criteria. A final ranking of criteria could have provided additional value. Furthermore, we changed the timing of evaluations: during the season in 2014A&B and after the season in 2015A. This meant that actual grain yields could be used for evaluation instead of visual judgement in the field. Combined with a presentation of average regional yields, this could have led to better-informed evaluations of yields. Finally, the re-design sessions proved to be useful for general feedback on the demonstrations from farmers, highlighting region-specific challenges and exchanging knowledge between stakeholders to improve the relevance of practices. However, some issues raised during the re-design sessions did not fit within the scope of a legume project. The problems of rats, for instance, would require an integrated strategy with a whole new range of stakeholders. The co-design process helped to flag such issues, which require follow-up in the future.

### 4.2 A relevant basket of options, for different types of farmers?

We hypothesized that the co-design process would lead to options that could not have been developed by either farmers or researchers alone. We found that the process broadened the scope of the evaluation of technologies from researchers' criteria, such as yield, to a wider range of variables including specific variety traits, marketability, availability of inputs and the ease of staking. Capturing these multiple perspectives helped to explain why farmers may not always prefer treatments that could give them the largest yield (Biggs, 1990; Douthwaite et al., 2001; Le Gal et al., 2011). The knowledge exchange between different stakeholders also resulted in enhanced relevance of the demonstrated options, including the introduction of new options. Notable contributions from farmers were the suggestion for cost reduction of the banana fibre and sisal string staking method and for risk and labour reduction related to the number of seeds per hole. Farmers also provided immediate feedback on the suggestion from researchers to rotate climbing bean with Irish potato: in their view, this practice would not do well in the area. The removal of the growing tip of the bean was an example of a practice suggested by farmers, although the practice was discarded again due to the poor performance. Researchers analysed yields, costs and labour demand of the different treatments in the demonstrations, to compare the results with their initial expectations and to discuss them during the evaluations and re-design sessions. The deviation between expectations and performance often led to additional research in or parallel to the demonstrations. Researchers also contributed with new varieties and practices, such as TSP fertilizer and the different staking methods, from their experience in other countries. Extension officers and NGO partners mainly had a role in sharing of good practices or advice, e.g. on pest and disease management. Even when this did not lead to modifications in the demonstrations, their contributions helped to answer questions from farmers on local issues. The co-design process therefore added value over only demonstrating a researcher best-bet combination of practices expecting to lead to the largest yields, but also over just supplying a climbing bean variety and leaving experimentation and adaptation entirely up to farmers (cf. Biggs, 1990; Sumberg et al., 2003). The joint discussions widened the range of options compared with what farmers or researchers could have achieved alone (Bos et al., 2009; Meynard et al., 2012; Salembier et al., 2018).

Our second hypothesis was that the co-design process would result in relevant options for different types of farmers given their specific resource constraints. Although we found differentiated preferences between groups of farmers, this was mostly related to their geographical context, such as the preference for certain varieties, the availability of inputs and staking materials, use of intercropping or local challenges like birds and rats. The development of options for farmers with different socio-economic backgrounds proved more difficult. The introduction of banana fibre and sisal string was expected to provide a low-cost alternative staking option specifically for poorer farmers. The co-design process showed, however, that farmers still found this staking method too expensive or labour intensive. Also, poorer farmers producing for home consumption found costs and labour more important aspects of technologies than wealthier farmers, and preferred the treatment without inputs. This shows the difficulty of finding a suitable alternative for resource poor farmers: they face multiple constraints in multiple production factors and have little room for manoeuvre (Franke et al., 2016; Tittonell et al., 2007). Only changes at the institutional level may really create opportunities for these farmers (Dogliotti et al., 2014; Schut et al., 2016).

Although we could not explain all differences in preference based on geographical, socio-economic or gender characteristics, the co-design process improved the visibility of the diversity of users, and ensured that options for farmers with different preferences were included in the demonstrations. Some farmers involved in the re-design sessions made comments like: *“staking should not be a problem for serious farmers”*, *“strings are just a last resort option”*, and a field assistant commented: *“farmers will find yield the most important”* and *“only some women will like variety Kabale local”*. These statements may reflect the preferences of wealthier, male farmers who often have a more visible presence in interactions with researchers (Cornwall, 2003; Pircher et al., 2013), but they neglect other perspectives. The disaggregated analysis of the evaluation of practices allowed the identification of a wider range of options for farmers with different preferences, such as the inclusion of multiple varieties, single input options (manure or TSP only) or management recommendation for farmers growing climbing beans in intercropping with banana.

### 4.3 Applicability of a co-design process in large-scale projects

Previous co-design studies were often conducted during intensive interactions with a limited number of farmers over a relatively long period of time (Dogliotti et al., 2014; Prost et al., 2018). In large-scale research-for-development projects such time and research capacity may not be available (Snapp et al., 2002). Our study therefore had the explicit aim to experiment with methods that apply principles of co-design in large-scale projects. The detailed interactions during the co-design process resulted in a concrete research output that can be used for out-scaling to similar areas: the basket of options for climbing bean cultivation developed during the process is applicable across the East African highlands. Through the option-by-context matrix, new initiatives could take these options as a starting point, select the most promising options and add to or refine the basket of options (Coe et al., 2014). Hence, in new areas, researchers can draw on these advanced options and experiences and do not need to apply the same intensive co-design process.

Although some methods applied in this study were time consuming, the basic principles of demonstrations, evaluations and re-design could be applied at a larger scale. Evaluation of demonstrations and suggestions for re-design could take place during field days. Evaluation criteria can be based on previous studies (Kamanga et al., 2014; Misiko, 2013; Vandeplas et al., 2010), and may only have to be identified jointly for new technologies or practices such as the staking methods (Bellon, 2001; Nelson and Coe, 2014). The collection of household information from farmers participating in the project would enable disaggregated analysis of preferences. Such steps could be considered as a ‘light’ version of the co-design process.

Still, preferences for and performance of technologies may be highly farmer- or site-specific, and not easily captured in scaling domains. In our study, this was reflected in the lack of consistent yield responses to practices, and in the diversity of preferences and inconsistent or unexplained relationships with household characteristics. Understanding such differences to allow for local fine-tuning and adaptation will still require more intensive interactions with farmers (Defoer, 2002; Dumont et al., 2017; Kraaijvanger et al., 2016).

Based on this study we therefore advocate a basket-of-options approach, i.e. providing farmers with a collection of practices (c.f. Table 2) that can be combined in a flexible manner. Guidelines about the relevant context of practices and suggestions on how to experiment with these practices can be made available for extension, e.g. through manuals and boundary tools such as the option-by-context matrix (Clark et al., 2016; Coe et al., 2014). This could support farmers' own experimentation and adaptation of options (Kraaijvanger et al., 2016; Le Gal et al., 2011; Prost et al., 2018). Such an approach provides a practical alternative for detailed, site-specific recommendations applicable to single communities, and one-size-fits-all recommendations that are irrelevant for the majority of farmers.

## 5 Conclusion

In this study we evaluated the usefulness of an iterative co-design process to generate a relevant basket of options for a diversity of farmers in the context of a large-scale project. The study showed how farmers use multiple evaluation criteria, of which yield is but one. The iterative process, including the implementation of and feedback on co-designed practices by farmers, allowed the generation and selection of more relevant technology alternatives. The involvement of multiple stakeholders broadened the range of options for experimentation. The process also revealed the diversity of preferences among users. To include this diversity in the design and selection of technology options, future projects could benefit from a ‘light’ version of the co-design process. Although the process as applied in this study was time-consuming, the basket of options developed can be applied across the East-African highlands with the option-by-context matrix as a starting point for out-scaling. The study also showed, however, that consistent recommendations for out-scaling based on household characteristics were difficult to identify. This strengthens the plea for a basket of options with flexible combinations of practices rather than narrowly specified technology packages. Finally, the co-design process showed the difficulty of developing options for poor farmers, as they are confronted with multiple, binding constraints. Technology development should therefore go hand in hand with institutional innovation to relieve constraints for these farmers.

## Appendix A Supplementary data

Supplementary material Image 1
