# Supplementary material for: Co-design of improved climbing bean production practices for smallholder farmers in the highlands of Uganda
Source: Agric Syst. 2019 Oct;175:1–12. doi: 10.1016/j.agsy.2019.05.003 (PMC6686619; doi:10.1016/j.agsy.2019.05.003)
Supplement: Supplementary file 1 — Supplementary material [file mmc1.docx]

## Supplementary materials

**Table S1:** Varieties, inputs, staking methods and other practices shown in demonstrations in the eastern and southwestern highlands of Uganda in seasons 2014A, 2014B, 2015A and 2015B

| Variety | Inputs | | | Staking method | | Other practice | | Region and season | | |  |
| --- | --- | --- | --- | --- | --- | --- | --- | --- | --- | --- | --- |
|  | Manure | TSP | DAP |  | |  | | Eastern | | Southwestern |  |
| Kabale local | - | - | - | Single stakes | | Row planting | | 2014A, 2014B | | 2014B |  |
| Kabale local | + | - | - | Single stakes | | Row planting | | 2014B | | 2014B |  |
| Kabale local | - | + | - | Single stakes | | Row planting | | 2014B | | 2014B |  |
| Kabale local | + | + | - | Single stakes | | Row planting | | 2014A, 2014B, 2015A, 2015B | | 2014B |  |
| Kabale local | + | + | - | Tripods | | Row planting | | 2014B | | 2014B |  |
| Katuna | - | - | - | Single stakes | | Row planting | | NA | | 2015A, 2015B |  |
| Katuna | - | + | - | Single stakes | | Row planting | | NA | | 2015B |  |
| Katuna | + | + | - | Single stakes | | Row planting | | NA | | 2015A, 2015B |  |
| NABE 10C | + | + | - | Single stakes | | Row planting | | 2015A, 2015B | | NA |  |
| NABE 12C | - | - | - | Single stakes | | Row planting | | 2014B, 2015A, 2015B | | 2014B, 2015A, 2015B |  |
| NABE 12C | + | - | - | Single stakes | | Row planting | | 2014B | | 2014B, 2015A, 2015B |  |
| NABE 12C | - | + | - | Single stakes | | Row planting | | 2014B, 2015A, 2015B | | 2014B, 2015A, 2015B |  |
| NABE 12C | - | + | - | Sisal strings | | Row planting | | 2015A, 2015B | | NA |  |
| NABE 12C | - | + | - | Tripods | | Row planting | | 2015A, 2015B | | NA |  |
| NABE 12C | - | - | + | Single stakes | | Row planting | | 2015A, 2015B | | NA |  |
| NABE 12C | + | + | - | Single stakes | | Row planting | | 2014B, 2015A, 2015B | | 2014B, 2015A, 2015B |  |
| NABE 12C | + | + | - | Sisal strings | | Row planting | | 2014B | | 2014B, 2015A, 2015B |  |
| NABE 12C | + | + | - | Tripods | | Row planting | | 2014B | | 2014B |  |
| NABE 12C | + | + | - | Single stakes | | Broadcasting | | NA | | 2015A, 2015B |  |
| NABE 12C | + | + | - | Single stakes | | Removing growing tip | | NA | | 2015A, 2015B |  |
| NABE 26C | - | - | - | | Single stakes | | Row planting | 2014A | 2015A | | |
| NABE 26C | + | + | - | | Single stakes | | Row planting | 2014A, 2015A | 2015A | | |
| NABE 26C | + | + | - | | Banana fibre ropes | | Row planting | 2014A | NA | | |
| NABE 26C | + | + | - | | Sisal strings | | Row planting | 2014A | NA | | |
| NABE 26C | + | + | - | | Tripods | | Row planting | 2014A | NA | | |
| Fe-enriched | - | - | - | | Single stakes | | Row planting | NA | 2015A, 2015B | | |
| Fe-enriched | - | + | - | | Single stakes | | Row planting | NA | 2015B | | |
| Fe-enriched | + | + | - | | Single stakes | | Row planting | 2015A, 2015B | 2015A, 2015B | | |

## S2: Developing an ‘attainment index’ for technology evaluation

“The attainment index is a measure of the extent to which the overall performance of a technology option meets the interests and needs of a farmer or group of farmers” (Bellon, 2001). Farmers first scored the importance of a number of criteria for technology evaluation, followed by the scoring of the performance of treatments on each of these criteria. The attainment index was calculated based on the logic outlined by Bellon (2001): criteria were scored as 1 = very important, 0.4 = somewhat important, and 0 = not important; the performance of treatments for each criterion was scored as 1 = good, 0.5 = medium, and -1 = poor. The score of 0.4 for “somewhat important” was given to produce the ordering shown in Table S2, following the assumption that it is more desirable to have an intermediate performance for a very important characteristic than to have a very good performance for a characteristic that is “somewhat important.” Scores for performance and criteria can be combined in a matrix that produces an ordinal scale from more to less desirable. For each cell in the matrix the scores were multiplied, to obtain a combined score ranging between 1 and -1 (Table S2).

**Table S2: Matrix of scores for attainment index**

| Performance | Importance of criteria | | |
| --- | --- | --- | --- |
|  | Very important (= 1) | Somewhat important (= 0.4) | Not important (= 0) |
| Very good (= 1) | 1 | 0.4 | 0 |
| Intermediate (= 0.5) | 0.5 | 0.2 | 0 |
| Poor (= -1) | -1 | -0.4 | 0 |

*Source: Bellon (2001)*

The combined scores for all criteria were added to generate an overall weighted score per treatment: the attainment index. As some farmers may have rated a larger number of criteria as important than others, the index was normalized and divided by a ‘perfect score’ – the score that would have been obtained if the treatment had scored ‘good’ on all relevant criteria (the sum of ‘very’ and ‘somewhat important’ scores).

**Table S3:** Average score for importance of evaluation criteria (1 = very; 0 = somewhat; -1 = not important) in the eastern (E) and southwestern (SW) highlands of Uganda in season 2015A, and household characteristics having a significant (*P* < 0.05) positive or negative relationship with this score (region where relationship was significant indicated in brackets). * indicates significant difference in importance of criteria between regions (*P* < 0.05).

| Category | Criteria | Score | | Household characteristic | Pos./neg |
| --- | --- | --- | --- | --- | --- |
|  |  | E | SW | (region) |  |
| *General* | Yield | 0.92 | 0.93 |  |  |
|  | Costs | 0.42* | 0.82* | Proportion income farming (E) | - |
|  |  |  |  | Farm size (SW) | + |
|  |  |  |  | Production orientation (SW) | - |
|  | Benefit/cost ratio | 0.79 | 0.75 | Production orientation (SW) | - |
|  | Labour | 0.87* | 0.55* |  |  |
| *Varieties* | Yield without fertilizer | 0.92* | 0.26* | Income from salary/pension/ remittances (E) | - |
|  |  |  |  | Proportion income farming (SW) | + |
|  | Yield with fertilizer | 0.87 | 0.82 | Age hh head (SW) | - |
|  |  |  |  | Farm size (SW) | - |
|  |  |  |  | Income from salary/pension/ remittances (SW) | - |
|  | Grain size | 0.92 | 0.85 |  |  |
|  | Grain colour | 1.00 | 0.83 |  |  |
|  | Marketability | 0.66* | 0.96* |  |  |
|  | Taste | 0.79 | 0.93 |  |  |
|  | Maturity time | 0.76 | 0.88 | Production orientation (SW) | + |
|  | Tolerance insects | 0.79 | 0.73 |  |  |
|  | Tolerance other pests | 0.87 | 0.62 |  |  |
|  | Resistance disease | 0.97 | 0.84 | Farm size (SW) | + |
|  | Suitability for climate | 1.00 | 0.89 |  |  |
| *Inputs* | Availability inputs | 0.95* | 0.52* |  |  |
| *Staking* | Ease of staking method | 1.00 | 0.96 |  |  |
| *methods* | Availability staking material | 1.00 | 0.57 |  |  |
|  | Strength of staking material | 1.00 | 0.88 | Age household head (SW) | - |
|  | Re-usability staking material | 1.00 | 0.98 |  |  |
